# Association between densities of adult and immature stages of *Aedes aegypti* mosquitoes in space and time: implications for vector surveillance

**DOI:** 10.1186/s13071-022-05244-4

**Published:** 2022-04-19

**Authors:** Maisa Carla Pereira Parra, Camila Lorenz, Margareth Regina Dibo, Bruno Henrique Gonçalves de Aguiar Milhim, Marluci Monteiro Guirado, Mauricio Lacerda Nogueira, Francisco Chiaravalloti-Neto

**Affiliations:** 1 Laboratório de Pesquisa Em Virologia, Faculdade de Medicina de São José Do Rio Preto, São José Do Rio Preto, SP Brazil; 2 Department of Epidemiology, School of Public Health, University of Sao Paulo, São Paulo, SP Brazil; 3 Entomology Laboratory, Agency for the Control of Endemic Diseases, São Paulo, SP Brazil; 4 Vector Laboratory, Agency for the Control of Endemic Diseases, São José Do Rio Preto, SP Brazil

**Keywords:** Mosquito, Entomological index, Breteau index, Bayesian analysis

## Abstract

**Background:**

Mosquito control is currently the main tool available to contain the spread of several arboviruses in Brazil. We have evaluated the association between entomological surveys of female adult *Aedes aegypti* and the Breteau index (BI) in space and time in a hyperendemic area, and compared the human resources costs required to measure each of these indicators.

**Methods:**

Entomological surveys were conducted between 2016 and 2019 in Vila Toninho, a neighborhood in the city of São José do Rio Preto, Brazil. Monthly records of collected mosquito specimens were made and then grouped by season.

**Results:**

Our findings showed that adult and immature mosquitoes are more related in time than in space, possibly due to differences in their habitats or in climate variables. Bayesian temporal modeling revealed that an increase in 1 standard deviation in the BI was associated with a 27% increase in the number of adult female mosquitoes when adjusted for climatic conditions. The cost of entomological surveys of adult mosquitoes was found to be 83% lower than the cost of determining the BI when covering the same geographic area.

**Conclusions:**

For fine-scale assessments, a simple measure of adult *Ae. aegypti* abundance may be more realistic than aquatic indicators, but the adult indices are not necessarily the only reliable measure. Surveying adult female mosquitoes has significant potential for optimizing vector control strategies because, unlike the BI, this tool provides an effective indicator for micro-areas within an urban region. It should be noted that the results of the present study may be due to specific features of of the study area, and future studies should analyze whether the patterns found in the study neighborhood are also found in other regions.

**Graphical Abstract:**

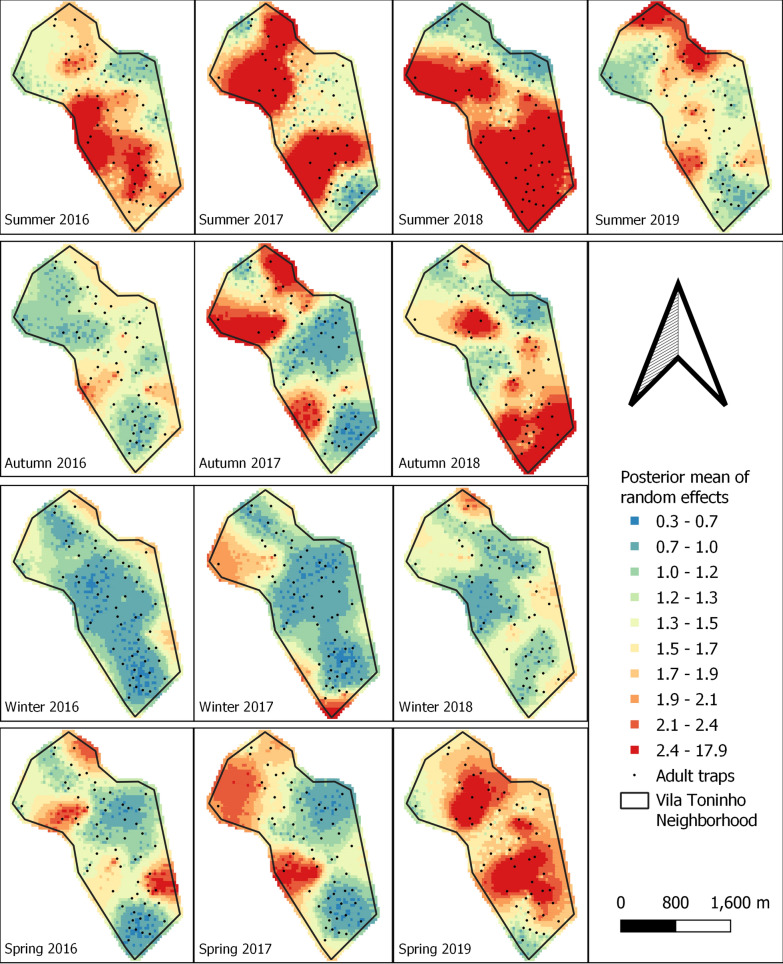

**Supplementary Information:**

The online version contains supplementary material available at 10.1186/s13071-022-05244-4.

## Background

*Aedes aegypti* is an invasive arboviral vector that transmits diseases with substantial global impact [1, 2] and which predominantly occupies urban and suburban areas associated with human populations [3, 4]. In Brazil, this mosquito is the primary vector of the viruses that cause dengue fever, chikungunya, Zika and urban yellow fever [5–10], and its presence has been reported in 5172 of the country’s 5494 municipalities [11].

Characterized by daytime blood-feeding behavior, female *Ae. aegypti* mosquitoes mainly lay their eggs in artificial containers where water collects and persists for many days [3, 12–14]. The presence of *Ae. aegypti* is also closely related to poor sanitation conditions and a lack of residual waste recycling facilities, which are typical of urban areas experiencing disorderly expansion. These two variables are considered to be significantly responsible for outbreaks of vector-borne diseases [15]. In Brazil, the main measure adopted for mosquito control is the elimination of habitats for immature stages [16]. *Aedes aegypti* abundance is also highly influenced by local environmental conditions that support larval development, resting sites and food and host access, especially when these conditions are all present within the 100-m flight range [17, 18].

Entomological surveillance, vector control and case monitoring are currently the only tools available to contain the dissemination of arboviruses. The most frequent indices to measure the abundance of *Ae. aegypti* are the block-level, quantitative indicators known as the Stegomyia indices, which evaluate immature forms of the mosquito under study (larvae and pupae). Nonetheless, questions remain regarding the ability of the Breteau index (BI), the house index or the container index to provide information on the production of adult mosquitoes [19–23] and, consequently, on the accuracy of these indicators to assess the real risk of arbovirus infection. BI is the relationship between the number of positive recipients and the number of properties surveyed; the house index is the ratio between the number of positive properties and the number of properties surveyed (percentage of houses positive for larvae); and the container index is the relationship between the type of positive container and the number of positive containers surveyed (number of positive containers per 100 houses) [49]. A promising alternative to monitoring vector infestation and arbovirus occurrence is the use of traps to collect adult mosquitoes. Several studies have shown that measuring the abundance of adult specimens can lead to the risk of arbovirus transmission being estimated with better precision [19, 24, 25] because adult females are the only category of mosquito involved in virus transmission.

In light of this situation, in this study we evaluated the association between surveying female adult *Ae. aegypti* and the BI over space and time in a hyperendemic area. We modeled the number of adult females in space and time to create vector infestation maps and also compared the cost of human resources required to collect adult mosquitoes to the cost of collecting the data required to determine the BI. We have interpreted the results to promote more effective risk-based arbovirus surveillance.

## Methods

### Study area

**Fig. 1:**
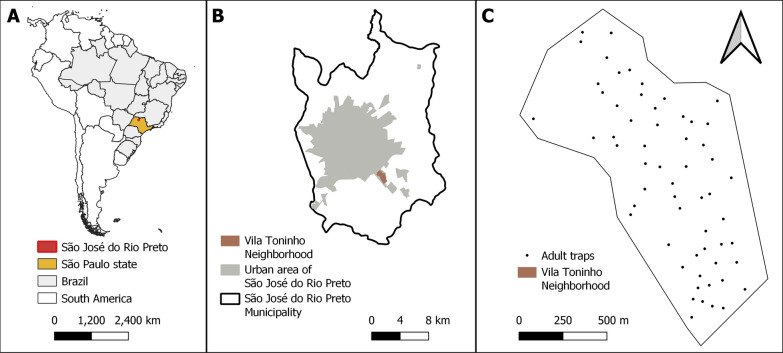
**a** Geographic location of São José do Rio Preto in São Paulo State, Brazil and South America. **b** Study area: the Vila Toninho neighborhood and its location in São José do Rio Preto. **c** location of the adult mosquito traps in the study area. The maps were built using QGis software 3.16.11 (https://www.qgis.org/)

This study was conducted in a neighborhood in the city of São José do Rio Preto, São Paulo State, Brazil between 2015 and 2019 [38, 41] as part of a larger cohort study on arbovirus occurrence in humans and vectors approved by the São Paulo Research Foundation (FAPESP; Grant Number 2013/21719-3). Re-introduction of *Ae. aegypti* mosquitoes into the municipality occurred in 1985 [42], and the first autochthonous case of dengue fever was confirmed in 1990. The study neighborhood, Vila Toninho (Fig. 1), is largely urban and is located in the southeastern area of the city. It has approximately 5600 inhabitants (density: 4800 per km^2^) [43] and 1940 households. Vila Toninho is located on the outskirts of São José do Rio Preto, and its socioeconomic indicators are poorer than those of the city as a whole. The mean income of heads of households in Vila Toninho is 1.9-fold that of the monthly minimum wage in Brazil, and 15.3% of households have ≥ 5 residents. Comparative values for the entire municipality are a mean income of 5.7-fold the monthly minimum wage, and 11.5% of households with ≥ 5 residents, respectively [43]. The study area has an undulating terrain and is characterized by dry winters with moderate temperatures (average: 21.9 °C) and wet summers with moderately high temperatures (average: 27.7 °C) [44].

### Data sources and entomological surveys

The procedures followed were based on Parra et al. [25]. Adult mosquitoes were captured using 30 BG Mosquitito™ traps (Biogents AG, Regensburg, Germany) that were positioned between December 2015 and February 2019. Traps were located outside of residences in the Vila Toninho neighborhood, close to plant pots and out of direct exposure to sunlight and rain, at preselected households that had shaded areas; collections were made 24 h after deployment. Traps were also installed in 30 houses, and the mosquitoes collected after 24 h; then the traps were redeployed in the same month but in 30 different houses. This process was performed monthly at the same households over the course of the study, which produced data from up to 60 households each month. The data from one trap were removed from the analyses due to a lack of consistency in the data, which meant that the final calculations are based on 59 traps. The Cartesian coordinates of these houses and individual traps (Data: WGS-84 and SIRGAS 2000) were obtained using the Global Positioning System (GPS). We attempted to cover the entire residential area of the neighborhood and distributed the traps according to the mosquito’s flight radius [17, 18, 26], maintaining an average distance of 149 m between each trap. Mosquitoes captured from the traps were identified at the Vector Laboratory of the Agency for the Control of Endemic Diseases based on taxonomic keys [45, 46]. We targeted adult female mosquitoes due their epidemiological importance. Temperature, precipitation and evapotranspiration data were obtained from a local database [47]. The modeling relied on seven climate variables: average daily precipitation, average minimum temperature, average maximum temperature, average temperature, average evapotranspiration, absolute minimum temperature and absolute maximum temperature. In the space–time analysis, the values of the climate variables were considered to be the same for all traps in each season. A previous study [38] showed that in small areas (such as the Vila Toninho neighborhood), there is no significant difference in climate measurements at each sampling point, as the traps are located relatively close to each other. In the temporal models, the values of the climate variables were considered monthly; in the spatiotemporal models, they were obtained and calculated by season.

To measure the BI we used a monthly one-stage larval sampling method, with blocks as primary sampling units [48]. We obtained a sample size of 600 households, considering an expected BI of 5.0% and a 95% confidence interval of between 2.6% and 7.4%. We increased the sample size to 1000 households (600 divided by 0.6), taking into account that 40% of them could be uninhabited or otherwise inaccessible. The blocks to be visited monthly were obtained by a random drawing with a weight proportional to the number of households. The larval survey was carried out on the selected blocks, with the exception of houses that were uninhabited or to which entry was denied by the resident [48].

Field agents assessed the number of larvae in all water containers present in each household surveyed. For each container with mosquito larvae, the agents collected a sample in a labeled glass tube that was sent for larval identification by the Vector Laboratory of the Agency for the Control of Endemic Diseases. To calculate the BI, we considered only those containers with *Ae. aegypti* larvae to be positive. The field agents wrote down the addresses of the surveyed households, and these were subsequently geocoded, which allowed us to obtain their Cartesian coordinates. To assess the BI, larval sampling was performed during the first 3 weeks of each month from December 2015 to February 2019 and preceding adult mosquito sampling, which occurred in the fourth week of each month. We adopted this procedure to provide the time lag necessary for larvae to develop into adult mosquitoes.

The BI is the relationship between the number of positive recipients and the number of properties surveyed, corrected for the result to be expressed as 100 properties [49]:

$${\text{BI = }}\frac{{{\text{Number of positive recipients for }}Ae. \, aegypti}}{{\text{Number of properties surveyed}}} \times 100.$$

In our study area, this number is usually very low, as positive recipients are temporary and have very little persistence over time. Thus, to achieve a representative sample, it was necessary to include a very large number of properties, as the vast majority of them would not have positive recipients (Additional file 1: Temporal model database).

To compare the cost-effectiveness of the two methods (one which relied on adult mosquito females and one which relied on immature stages), we considered time spent, the number of households visited per month and the number of field workers required to implement each method. To cover 1 month in the Vila Toninho neighborhood, the workers spent the first 3 weeks of each month performing the BI method and the last week of the month performing the adult survey. We estimated the cost-effectiveness using the “man-day” variable, which was calculated by multiplying the number of days worked by the number of workers and the number of teams required to measure each index.

### Data analysis

In the temporal analysis, we modeled the number of *Ae. aegypti* adult females taking into consideration only a temporal architecture. We evaluated the relationship between the total number of adult female mosquitoes collected by adult traps in the study area each month from December 2015 to February 2019 as a function of the BI, as well as for the entire study area and by month. We also considered climate as a variable in our models and adjusted accordingly to determine the true relationship between the number of adult mosquitoes and the BI. Because we modeled a temporal series, the model included a temporal random effect (autoregressive correlation of order 1 [AR1]), as supported by Zuur et al. [50]. We present the expressions of this model in Additional file 2: Model expression. Because the model included climate variables, we chose the best temporal model using the deviance information criterion (DIC) [48].

In order to assess the spatiotemporal association between the number of adult mosquitoes and BI, we group their values into quarters representing the four seasons. This step was included to account for the seasonality of the vector, which presents higher infestation rates in the summer months (December to February). This aggregation was also necessary for increasing the size of the samples to measure BI. We incorporated the Cartesian coordinates of the traps and households surveyed in a Geographic Information System. We then created 150-m buffers around each trap and considered the household surveyed for the measurement of BI within each buffer and during each quarter-year. The distance of 150 m was based on the mosquito’s flight radius [17, 18, 26]. We obtained the BI values by trap and quarter considering the number of containers positive for immature *Ae. aegypti* and the households surveyed within each buffer and quarter.

Adult mosquito modeling based on traps and quarter-year periods was performed using a model with a spatiotemporal correlation (AR1) in which the dependent variable was the number of adult mosquitoes (adults) and a Poisson probability distribution was assumed. Initially, only an intercept model with a spatial random effect correlated temporally, and an independent and identically distributed (*iid*) random effect was considered, as shown in Additional file 2.

The spatiotemporal random effects were composed of a temporal component and a spatial component. We used AR1 as the temporal component. The spatial component was defined as the spatial dependence between the locations where the traps were installed. This was modeled by* W*, the realization of the latent stationary Gaussian field.* W* was obtained from the Euclidean distances between the places where the traps were installed and considering a Matérn function [51] and stochastic partial differential equations. It was represented by a Markovian Gaussian random field [50] built on triangle meshes. The purpose of these spatiotemporal models is to consider that the spatial random field can vary over time and to do so using AR1 to address their temporal portion [50, 52, 53]. We included the independent and identically distributed random effect to account for the use of the same households to install the traps during the study period.

We obtained exponentiated values of the spatial random effects for all vertices of the mesh organized by quarter-year. In order to obtain a representation of these effects in the entire study area, the IDW algorithm (the inverse of the distance) was used on each quarter-year period to interpolate the values of the random effects for a grid of 10,000 points. In each specific location of the study area and in each quarter-year, the spatial random effects represented how much higher the number of *Ae. aegypti* females was (in the case of values above the unit) or how much lower the number of *Ae. aegypti* females was (in the case of values below the unit) than the estimated average number over the entire study period. These values were considered in the model, as were the respective numbers of adult mosquitoes (also organized by buffers and quarter-year periods). After these BI values were obtained, they were included in the models that considered the spatiotemporal and *iid* random effects. As in the temporal models, these models included climate variables to more accurately establish the relationship between the number of adult mosquitoes and the BI, as presented in Additional file 2.

The temporal and spatiotemporal models were created in a Bayesian context using the integrated nested Laplace approximation (INLA) approach [54]. We used non-informative priors for the fixed effects and priors with penalized complexity for the random effects [55].The R software (R Core Team 2019; R Foundation for Statistical Computing, Vienna, Austria) and the R-INLA package (www.r-inla.org) were used to perform the modeling. The QGIS software (QGIS Development Team 2021) was used to build the maps.

## Results

Between December 2015 and February 2019, our traps captured 1169 adult *Ae. aegypti* females in the Vila Toninho neighborhood (Fig. 1). Vila Toninho is a poor neighborhood that has suffered dengue outbreaks for several years. In recent years, several epidemiological and entomological studies have been performed there, as have educational campaigns to reduce the number of breeding sites in homes. The low number of mosquitoes found here is likely the result of these campaigns.

**Fig. 2:**
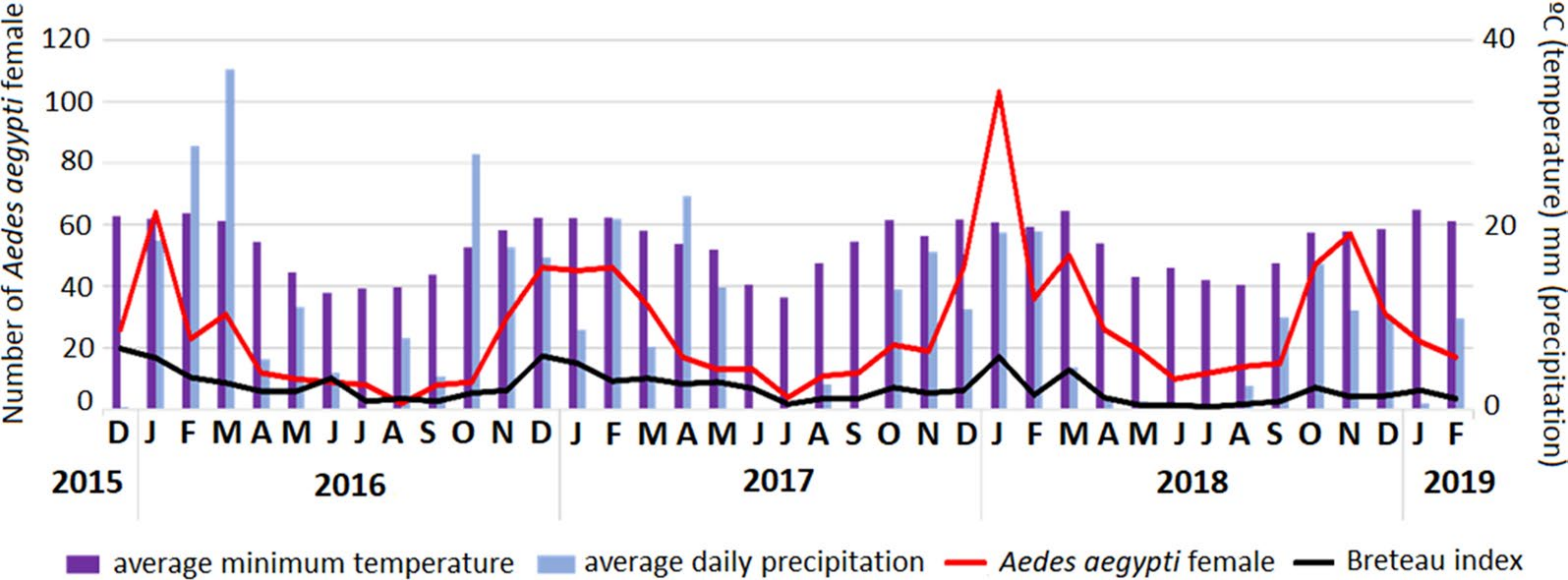
Average daily precipitation (mm), average minimum temperature (°C), number of *Aedes aegypti* adult females and Breteau index over time. Study location was the Vila Toninho neighborhood in São José do Rio Preto, São Paulo State, Brazil, and sampling occured between December 2015 and February 2019

**Fig. 3:**
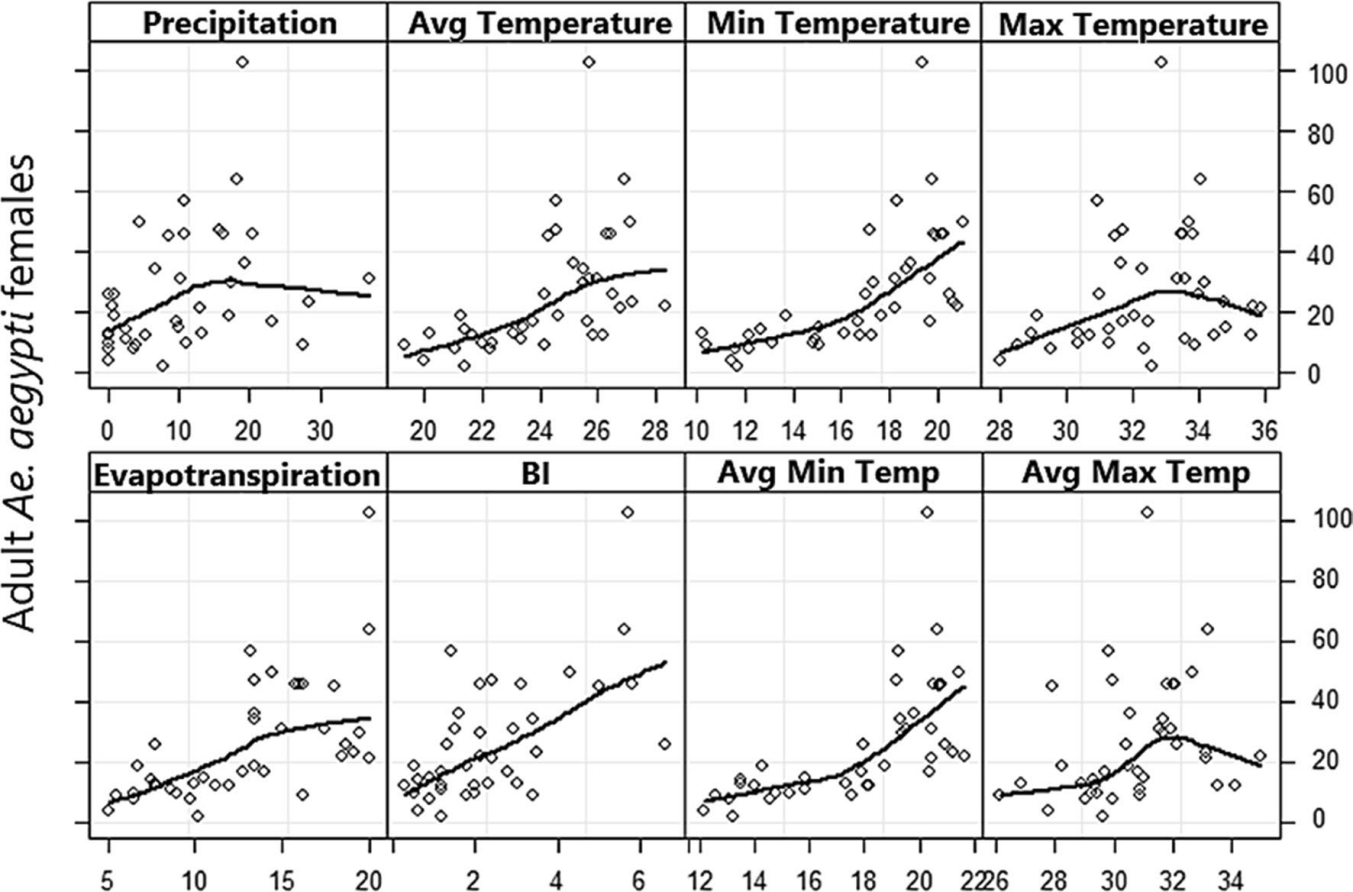
Scatterplots with locally weighted smoothing lines showing the relationship between climate variables and adult *Ae. aegypti* females in the Vila Toninho neighborhood of São José do Rio Preto between October 2015 and February 2019. Abbreviations: Avg, average; BI, Breteau index. The graphs were built using R Core Team software 1.3.1093 (https://www.R-project.org)

In addition, an average of 1126 households were sampled during the study, and 757 (about 70%) households were evaluated each month to search for recipients that tested positive for the presence of immature *Ae. aegypti* in order to measure the BI. We missed, on average, 32.8% of the residences because they were uninhabited or because residents did not allow the field agents to enter. This value was lower than the 40.0% considered to be lost in our sampling plan. Figure 2 shows the number of adult female mosquitoes, the BI and the climate variables over the 39 months of the study period. These indicators were found to be clearly influenced by both temperature and periods of rain and drought. Details on the relationship between each specific variable and the number of adult mosquitoes is shown in Fig. 3. The values of these variables are presented in Additional files 1 and 3.

All variables related to temperature, including evapotranspiration, were collinear with each other. Thus, for modeling, we chose only the one with the best DIC in the bivariate models in order to determine whether the positive relationship between adult mosquito presence and the BI remained significant even after adjusting for climate variables. The final covariables used in the temporal models were BI, average daily precipitation and average minimum temperature (Table 1). We used standardized variables (subtraction of the mean and division by standard deviation [SD]) and considered an AR1 temporal random effect in these temporal models. An increase of 1 SD in the BI was associated with a 46% increase in the number of adult mosquitoes when the results were not adjusted for climate variables. After adjusting for climate, an increase of 1 SD in the BI was associated with a 27% increase in the number of adult female mosquitoes. The best temporal model obtained (the lowest DIC) relied on the BI and average minimum temperature. The lack of significance of rainfall in this model may be due the lack of statistical power of the temporal sample size to identify a significant relationship with the number of mosquitoes.

**Table 1:** Posterior mean fixed effects and 95% confidence intervals for the number of adult *Aedes aegypti* females in temporal modeling in the Vila Toninho neighborhood of São José do Rio Preto, São Paulo State, Brazil

Model	Covariate	Mean	95% CI	DIC
Intercept		26.12	24.54–27.73	711.9
Intercept + AR1		21.80	10.34–40.63	257.1
BI + AR1	Intercept	20.56	10.88–34.13	255.3
	BI	1.46	1.22–1.72	
Min Temp + AR1	Intercept	20.01	14.76–25.76	255.9
	Min Temp	1.78	1.43–2.16	
Precip + AR1	Intercept	22.23	10.14–42.66	258.0
	Precip	1.16	0.95–1.40	
BI + Min Temp + AR1	Intercept	19.78	12.85–27.28	254.9*
	BI	1.27	1.06–1.50	
	Min Temp	1.51	1.19–1.89	
BI + Precip + AR1	Intercept	21.11	9.73–38.20	256.6
	BI	1.46	1.24–1.71	
	Precip	1.17	0.99–1.35	
BI + Min Temp + Precip + AR1	Intercept	19.86	12.16–28.68	255.8
	BI	1.29	1.07–1.53	
	Min Temp	1.44	1.11–1.84	
	Precip	1.09	0.92–1.26	

AR1, Temporal random effect; BI, Breteau index; CI, confidence interval; DIC, deviance information criterion; Min Temp, average minimum temperature; Precip, average daily precipitation

*Best DIC

All temporal autocorrelation correlograms of the models presented in Table 1 can be seen in Additional file 4: Temporal autocorrelation correlograms and random effects. The intercept model presented temporal autocorrelation in several lags and, after the introduction of the AR1 temporal random effect, the autocorrelation remained only in lag 1. The same findings occurred with the model that we considered to be the best (BI, average minimum temperature and AR1 temporal random effect). The exponentiated posterior means of the temporal random effects of the model with intercept and our final model are also presented in Additional file 4. There, our final model shows that the temporal autocorrelation present in our response variable was well explained until December 2017. Nonetheless, from January 2018 onward, some temporal autocorrelations remained unexplained.

The objectives of the spatiotemporal modeling performed herein were to evaluate the relationship between the number of adult female mosquitoes and the BI, and to map out the number of mosquitoes in space and time, with adjustments for spatial and temporal autocorrelations and climate (Table 2). To achieve this, we considered temporally correlated spatial random effects (AR1) and an independent and identically distributed random effect. The model in which only BI was considered exhibited a worse fit than the model in which only the intercept and random temporal and spatial effects were applied. The former model found that an increase of 1 SD in the BI would be associated with a 12% increase in the number of adult females. This result is weaker than that of the temporal model, in which an increase of 1 SD in the BI was associated with a 46% increase in the number of females (without adjusting for the climate variables). In the models in which the BI was adjusted for climate variables, the BI became non-significant (Table 2), suggesting that there is no relationship in space between the two entomological indicators. The database we used for running the spatiotemporal models is presented in Additional file 3: Spatiotemporal model database.

**Table 2:** Posterior mean fixed effects and 95% CI for the number of adult *Ae. aegypti* females in spatiotemporal modeling performed on the Vila Toninho neighborhood

Model	Covariate	Mean	95% CI	DIC
Intercept		1.33	1.25–1.41	2991.2
Intercept + RE		0.87	0.70–1.07	2264.9
BI + RE	Intercept	0.87	0.70–1.06	2266.7
	BI	1.12	1.00–1.24	
Min temp + RE	Intercept	0.82	0.69–0.96	2230.6
	Min temp	1.71	1.52–1.91	
Precip + RE	Intercept	0.86	0.69–1.06	2238.8
	Precip	1.53	1.35–1.73	
BI + min temp + RE	Intercept	0.82	0.69–0.95	2232.2
	BI	0.99	0.89–1.09	
	Min temp	1.72	1.52–1.94	
BI + Precip + RE	Intercept	0.88	0.70–1.08	2235.2
	BI	1.07	0.96–1.18	
	Precip	1.51	1.34–1.71	
BI + min temp + precip + RE	Intercept	0.82	0.68–0.97	2225.4*
	BI	0.99	0.89–1.09	
	Min temp	1.58	1.36–1.83	
	Precip	1.16	1.00–1.33	

RE, Random effects

*Best DIC

**Fig. 4:**
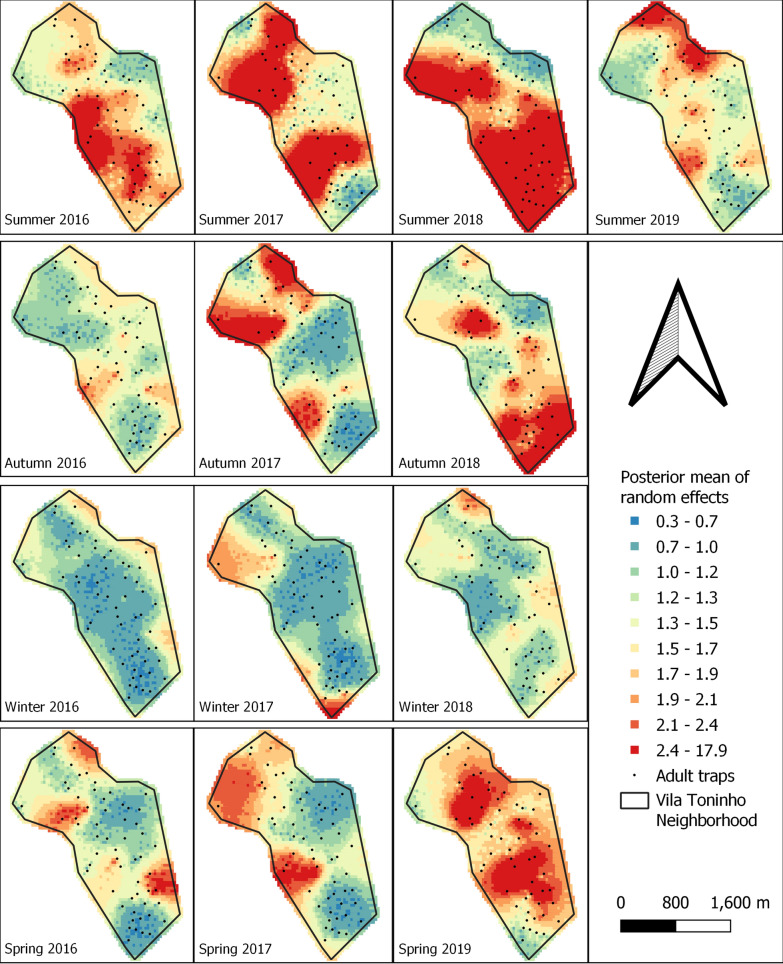
Posterior means of the spatial random effects for the spatiotemporal modeling of adult *Ae. aegypti* females per season and year in the Vila Toninho neighborhood. The maps were built using R Core Team software 1.3.1093 (https://www.R-project.org)

Figure 4 shows the infestation rates of adult female mosquitoes in space and time. The *Ae. aegypti* adult female infestation levels presented in seasons and years in these maps accompany their temporal trend (Fig. 2). In addition to identifying this temporal trend, we can also use the maps presented in Fig. 4 to distinguish between the levels of adult infestation in different small local areas within the Vila Toninho neighborhood.

The cost-effectiveness of these two entomological indices was also considered. Table 3 summarizes the main information on the composition of field teams, the time required and the number of households visited per month. Measuring the BI was found to require threefold more time than conducting the surveys of adult mosquitoes—when surveying covered the same area (the Vila Toninho neighborhood). The cost of entomological surveys of adult mosquitoes was also found to be 83% lower than the cost of determining the BI over the same geographical area.

**Table 3:** Differences in cost-effectiveness between two entomological indices (the BI and adult mosquito surveying via specimen capture) applied to cover the same area: the Vila Toninho neighborhood

Entomological index	Composition of field teams	Time required to perform each method per month	Number of households visited per month	Man-days^a^
Breteau index	1 driver and 3 field workers	3 weeks	757 households	120
Adult survey	1 driver and 1 field worker	1 week	59 households	20

^a^The man-day was calculated by multiplying the number of days worked by the number of workers and the number of teams required to measure each index

## Discussion

The principal finding of this study is that adult female mosquitoes and the BI are related temporally and that there is a weakened relationship in space. This divergence between two entomological indicators in space may be due to either mosquito larvae, pupae and adults requiring specific habitat characteristics or because of the existence of indoor breeding sites. Getis et al. [26] reported relevant divergences in the spatial composition of adult mosquito populations compared to that of immature populations. Several other factors, including high larval mortality, the brief lifespan of larvae and pupae and a short data collection period could result in immature abundance measurements that do not always correspond in space with the biologically relevant adult estimators [26, 27]. In terms of block-level indicators, aggregating household data may skew the calculation if the distribution of immature counts were to be concentrated in only a few households. Block-level estimators, such as the BI and the house index, the latter categorizing containers or households as “infested” if any larvae or pupae are found, may not represent the contribution of container productivity. Pupae-per-person and pupae-per-hectare measurements are sensitive to bias from inaccuracies in population data or area data, as well as to sampling error caused by the short duration of the pupal life stage [28].

The *Ae. aegypti* populations in the Vila Toninho neighborhood differed significantly between seasons, similar to findings reported in other regions [25, 27, 33]. We argue that it is possible to precisely estimate the infestation of adult female mosquitoes in small intra-urban areas by optimizing control and surveillance measures. Unlike traditional measurements, which are recorded at the city or neighborhood levels, the major strength of adult female mosquito surveys is the resulting higher resolution in time and space [34]. In our study, we analyzed an average of 59 traps (adults) and 757 houses (BI). Although BI apparently has a higher spatial resolution because of its requirement that more houses be visited, it is a block-level indicator. Its value is usually very low, as positive recipients are temporary and have very little persistence over time. Thus, a highly representative sample must include many properties, as the vast majority of these will not have positive recipients.

On the other hand, the collection of adult mosquitoes does not require substantial sampling effort for the same geographic area, since the individuals are winged and can travel a given flight radius to the traps. A large number of low-cost traps can be used in a longitudinal monitoring survey. Depending on operational needs, the index can be estimated at different spatial scales. Data can be aggregated to evaluate the *Ae. aegypti* abundance in the country or analyzed separately to estimate the population within a predefined region [34]. Some studies have shown that surveillance of adult *Aedes* mosquitoes would therefore be more suitable for arbovirus risk assessment than measures of immature stages [25, 34] since the association between larval indices and dengue transmission has yet to be proven satisfactorily [35]. Despite reductions in infestation levels in the winter, a sufficient number of mosquitoes survive to preserve the residual population until propitious climatic conditions occur the following spring; this phenomenon could maintain the cases of dengue, Zika and chikungunya in the region even during the coldest and driest period of the year, as observed in other regions [36].

Regarding the temporal models, an increase of 1 SD in BI was associated with a 46% increase in the number of female mosquitoes. When we adjusted for temperature or for temperature and precipitation, this value decreased to 27% (or 29%, respectively) but remained significant. This finding suggests that there is a relationship between adult mosquitoes and the BI that is independent of the climate. However, the spatiotemporal model revealed a lower correlation between the two entomological indicators: the regression coefficient decreased and remained at the limit of significance (mean: 1.12; 95% CI: 1.00–1.24). When we adjusted for climate variables, the BI lost its significance. The seasonal variations in the region influenced adults and immatures in the same way, so some correlation between them was found only over time, but not in space. Eisen et al. [29] showed a positive linear relationship between water temperature and the developmental rate of *Ae. aegypti* larvae between 15 °C and 30 °C. Seasonal temperatures between 20 °C and 31 °C can modify the metabolic rate of mosquitoes, shorten the period of larval development and optimize foraging and egg-laying behavior, thus leading to higher mosquito infestation when suitable immature habitats are available [30–32]. Therefore, both adult and larval abundances should be associated with each other over time.

Our results show that calculating the larval entomological index is more expensive than using adult traps because the BI methodology requires relatively more field agents and more time to cover the same geographic area. The cost in man-days to perform an entomological survey of adult mosquitoes is 83% lower than the cost required to determine the BI. Although the costs of traps (about US$100 each) and batteries (US$20 each) also have to be considered, given the large difference in productivity, this additional cost would be offset in the medium term. For example, if the annual budget were to be US$10,000, and the cost in man-days to collect adult mosquitoes is 83% less than that to determine the BI, then only US$1700 would need to be spent on field workers. Even if the cost of each trap were to be added to the budget, together with the cost of its respective battery (100 + 20 = US$ 120), and 60 traps were needed to cover the area, the cost would be US$8900. This strategy would be cost-effective because traps and batteries only need to be acquired once. Thus, in upcoming years money from the budget would only be spent on field workers, which would be much lower that the amount needed for the BI measure. The great advantage to capturing adult mosquitoes is that, unlike the BI or similar indices, this type of survey allows for a local assessment of the infestation. Only a few households are tested when the aim is to measure the BI, and this index is then taken to be representative of a larger area. There is an incorrect but commonly held belief that adult mosquito measurement is time-consuming, laborious and/costly [35]. For this reason, a large majority of studies have focused on immature forms of the mosquito, but immature forms of mosquitoes may not be the most efficient indicator for assessing disease risk [27, 35].

The strengths of our study include the 4-year surveillance period, the emphasis on adult female mosquitoes, the consideration of climate variables in the model, and the spatiotemporal approach. The models used were in accordance with the basic assumptions of regression modeling since they explicitly incorporated parameters representative of the spatial and temporal autocorrelation present in the response variables. Our study also has limitations in terms of the results of our temporal and spatiotemporal models. The results consider the remaining temporal autocorrelation of the residuals in lag 1, and the temporal autocorrelation remained unexplained from January 2018 onward due to covariates not being included in our final temporal model. We found patterns in adult mosquito abundance rates that persisted over time, and these patterns may be explained by covariates not included in our model. Consequently, future studies should add geographic, socioeconomic and other climate variables to these models, such as land cover acquired via satellite images [37, 38] and El Niño Southern Oscillation data, for example. There is also the question of representativity and comparability in pre-selecting houses for installing adult traps. There was no such pre-selection of houses for methods for determining the BI. The intrinsic characteristic of the BI method is the random choice of properties in a block. The adult traps also had their share of randomness, as they were installed on those properties that residents allowed, and not strictly on those that were chosen a priori. Another possible limitation is that the spatiotemporal model couldn not be used monthly because the number of mosquitoes caught per month was very small, as was the monthly BI of houses in the buffers. We therefore decided to pool the seasons to increase this number. Although the acuity of the two methods is different, we believe that it would not change the final relation between both indexes, since the months within each season in the study area present very similar characteristics in relation to climate variables.

The general expectation was that the abundance rates of larvae and adults would be related in space, but we did not find this association. It is worth mentioning that the results of the present study may be due to specific features of the study area, and future studies should analyze whether the patterns found in the study neighborhood are maintained in other regions. It is possible that Vila Toninho is particularly heterogeneous in terms of its land use and that the mosquito population is changing on a much finer spatial scale was measured in the present study. Another possibility is that indoor habitats (or other habitat types not recorded in the BI) are serving as habitats for immatures. A larger set of complex data with different approaches is needed to determine whether the temporal and spatiotemporal relationships found in the present study are consistent with findings reported from other areas [39]. Algorithms using artificial intelligence and deep learning have shown promising results when dealing with big data and could be tested on infestation data from both adult and immature mosquitoes [40].

## Conclusions

Adult and immature *Ae. aegypti* mosquitoes were found to be more closely related in time than in space. The temporal relationship between both indicators persisted even when the findings were adjusted for climate variables, and this consistency may be related to the biology of the mosquito vector in its different life stages. Bayesian temporal modeling revealed that an increase in 1 SD in the BI was associated with a 27% increase in the number of adult female mosquitoes when adjusted for climatic conditions. However, the relationship determined between adults and immatures in space was not significant and may have resulted from differences in their habitats. *Aedes aegypti* populations in the neighborhood studied in the present study were found to vary by season; however, even surveys carried out in winter revealed sufficient numbers of mosquitoes to sustain the residual population until environmental conditions become favorable in the spring. Calculating the larval entomological index was found to be more expensive than using adult traps, since the BI method requires relatively more field agents and more time to cover the same geographic area. Our results have relevant implications for *Ae. aegypti* mosquito control in Brazil. This study provides evidence that estimating adult mosquito infestation may be the best option, in terms of cost, efficacy and the ability to achieve a higher resolution in time and space. It is worth mentioning that the results reported here may be due to specific features of the study area, and future studies should analyze whether the patterns found in the study neighborhood are maintained in other regions. Further work is now needed to apply the associations identified to larger areas and to consider them in different socioeconomic contexts.

## Supplementary Information

**Additional file 1. **Temporal model database.

**Additional file 2. **Model expression.

**Additional file 3. **Spatiotemporal model database.

**Additional file 4. **Temporal autocorrelation correlograms and random effects.

**Additional file 5. **Script for temporal model in R software.

**Additional file 6. **Script for spatiotemporal model in R software.

## Data Availability

All data generated or analyzed during this study are included in this published article (and its Additional files). The codes of the temporal and spatiotemporal models are shown in Additional files 5 and 6, respectively.

## Abbreviations

AR1: Autoregressive correlation of order 1
DIC: Deviance information criterion
BI: Breteau index
