# Supplementary material for: Association between densities of adult and immature stages of Aedes aegypti mosquitoes in space and time: implications for vector surveillance
Source: Parasit Vectors. 2022 Apr 19;15:133. doi: 10.1186/s13071-022-05244-4 (PMC9020056; doi:10.1186/s13071-022-05244-4)
Supplement: Supplementary file 2 — Additional file 2. Model expression. [file 13071_2022_5244_MOESM2_ESM.pdf]

Supplementary Material 2 - Correlograms for the temporal models presented in Table 1 and the temporal random effects

The model only with intercept presented temporal autocorrelation in lags 1,5,6,7 and 11 (Figure 1). After the introduction of the AR1 temporal random effect in the model, the temporal autocorrelation remained only in lag 1 (Figure 2). The model with AR1 temporal random effect and Breteau index presented temporal autocorrelation in lags 1 and 5 (Figure 3). The model with AR1 temporal random effect and average minimum temperature was the only one that did not presented temporal autocorrelation (Figure 4). All the others models remained with temporal autocorrelation in lag 1, including that we considered our best temporal model, which included Breteau index and average minimum temperature (Figures 5 to 8).

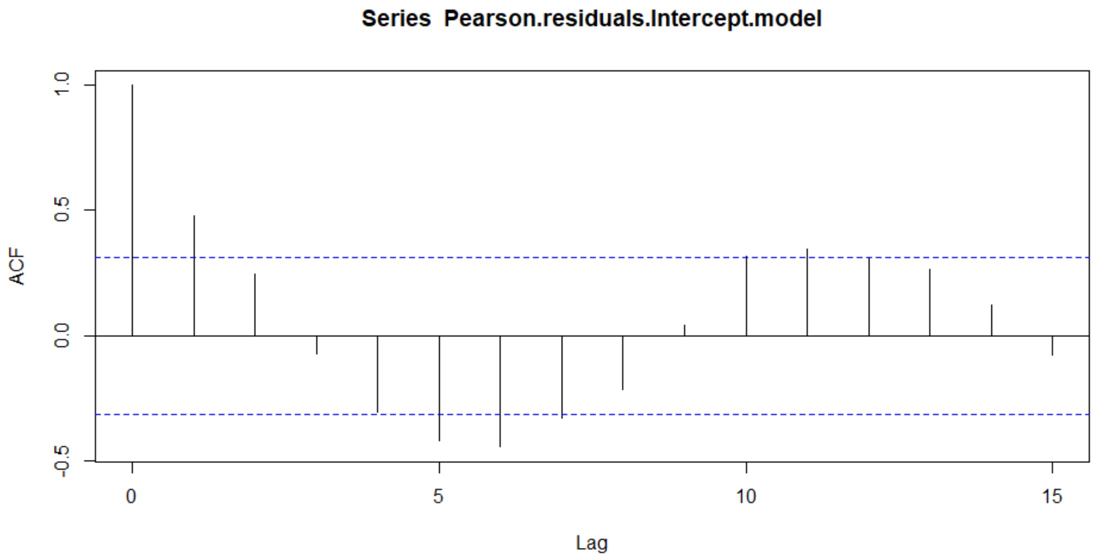

Figure 1 – Correlogram of the residual of the model with only the intercept in temporal modeling in the Vila Toninho neighborhood of São José do Rio Preto, São Paulo State, Brazil.

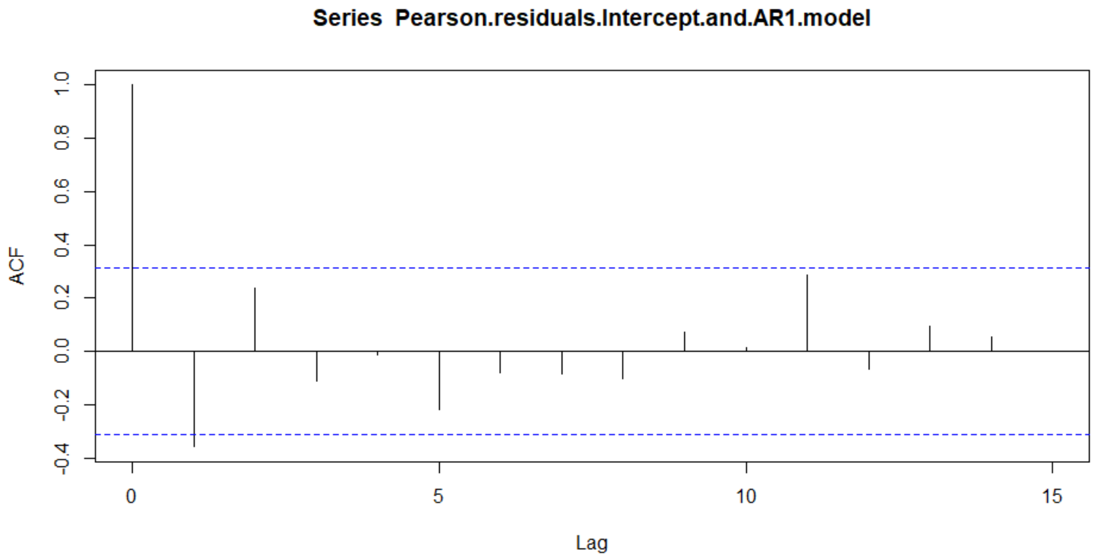

Figure 2 - Correlogram of the residual of the model with only the intercept and AR1 temporal random effect, in temporal modeling in the Vila Toninho neighborhood of São José do Rio Preto, São Paulo State, Brazil.

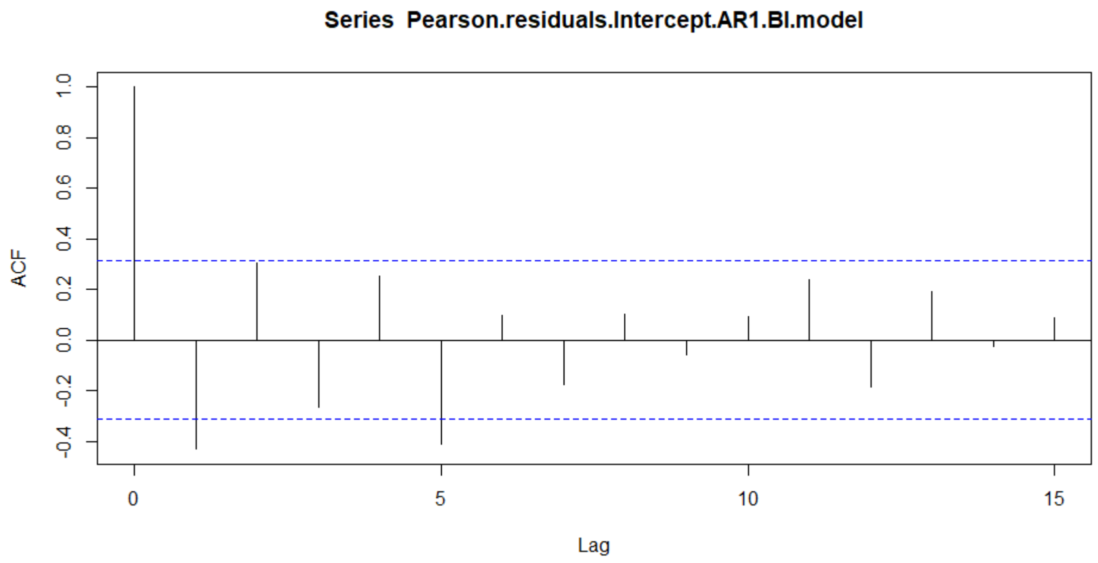

Figure 3 - Correlogram of the residual of the model with only the intercept, AR1 temporal random effect and Breteau index, in temporal modeling in the Vila Toninho neighborhood of São José do Rio Preto, São Paulo State, Brazil.

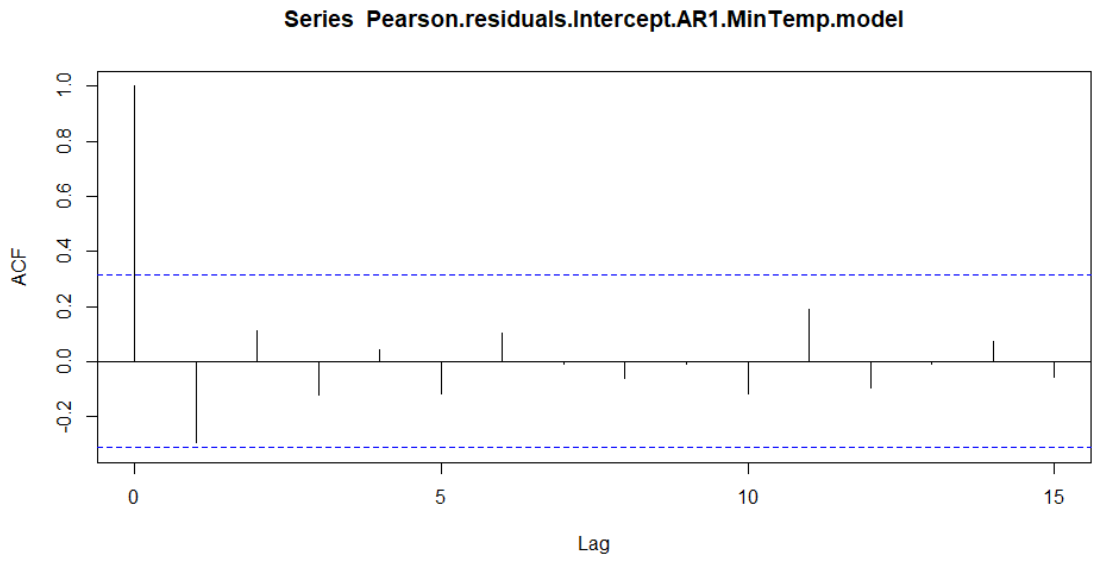

Figure 4 - Correlogram of the residual of the model with only the intercept, AR1 temporal random effect and average minimum temperature, in temporal modeling in the Vila Toninho neighborhood of São José do Rio Preto, São Paulo State, Brazil.

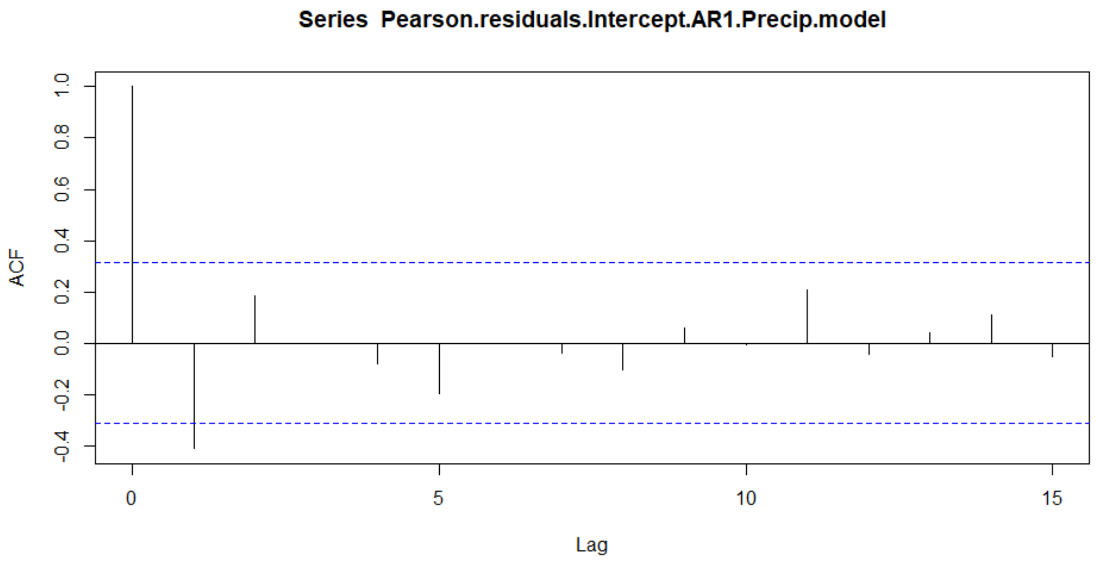

Figure 5 - Correlogram of the residual of the model with only the intercept, AR1 temporal random effect and average daily precipitation, in temporal modeling in the Vila Toninho neighborhood of São José do Rio Preto, São Paulo State, Brazil.

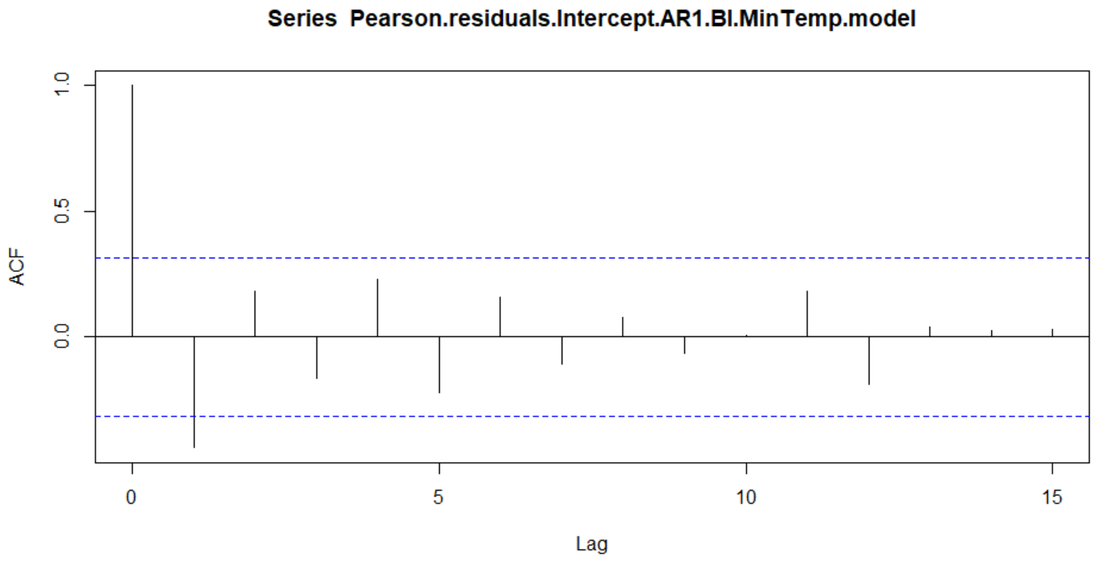

Figure 6 - Correlogram of the residual of the model with only the intercept, AR1 temporal random effect, average minimum temperature and Breteau index, in temporal modeling in the Vila Toninho neighborhood of São José do Rio Preto, São Paulo State, Brazil.

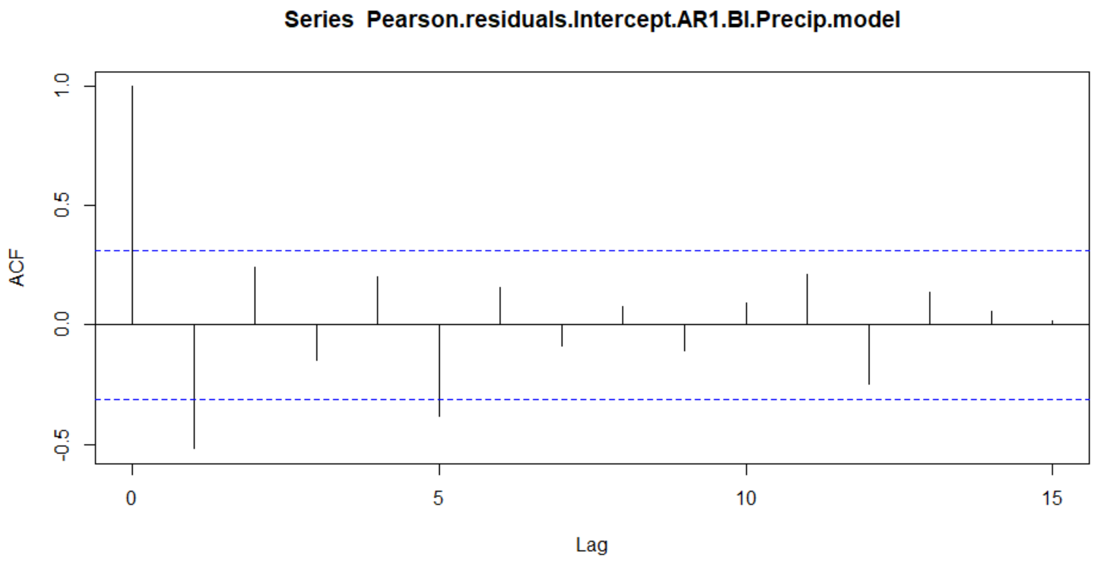

Figure 7 - Correlogram of the residual of the model with only the intercept, AR1 temporal random effect and average daily precipitation and Breteau index, in temporal modeling in the Vila Toninho neighborhood of São José do Rio Preto, São Paulo State, Brazil.

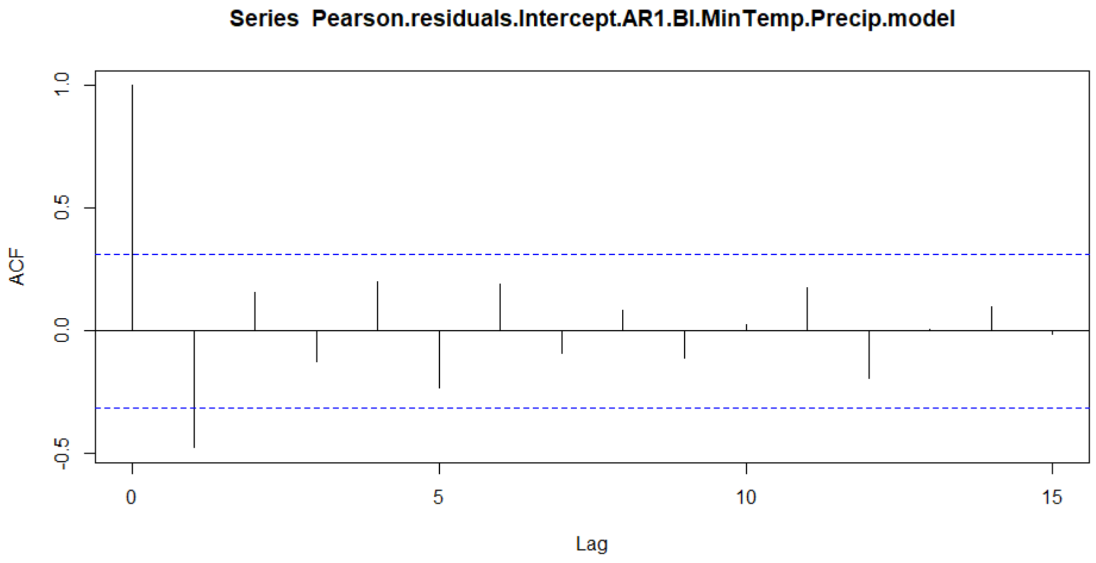

Figure 8 - Correlogram of the residual of the model with only the intercept, AR1 temporal random effect, average minimum temperature, average daily precipitation and Breteau index, in temporal modeling in the Vila Toninho neighborhood of São José do Rio Preto, São Paulo State, Brazil.

Figure 9 presents the temporal random effects (exponentiated) of the model with intercept and AR1 and our final model (intercept, AR1, Breteau index and average minimum temperature). When we compare these two curves, it is possible to see, in our final model, that the temporal autocorrelation present in our response variable (number of adult *Aedes aegypti* female) was well explained until December 2017. From January 2018 onwards, some temporal autocorrelation remained not explained, due to covariates not included in our final model, which we considered another limitation of our study.

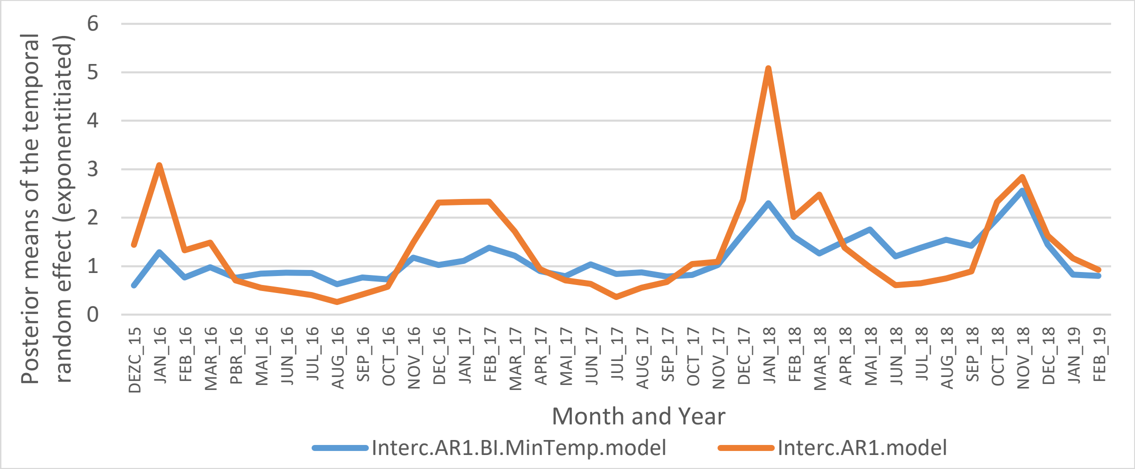

Figure 9 – Posterior means of the temporal random effects (exponentiated) of the model with intercept and AR1 (orange line)) and the model with intercept, AR1, Breteau index and average minimum temperature (blue line), Vila Toninho neighborhood of São José do Rio Preto, São Paulo State, Brazil, December 2015 to February 2019.
