# Supplementary material for: Association between densities of adult and immature stages of Aedes aegypti mosquitoes in space and time: implications for vector surveillance
Source: Parasit Vectors. 2022 Apr 19;15:133. doi: 10.1186/s13071-022-05244-4 (PMC9020056; doi:10.1186/s13071-022-05244-4)
Supplement: Supplementary file 4 — Additional file 4. Temporal autocorrelation correlograms and random effects. [file 13071_2022_5244_MOESM4_ESM.pdf]

Supplementary Material 4 – Expressions of our models

1 – Temporal model

$$Adult\ mosquitoes_t = Poisson(\mu_t)$$

$$log(\mu_t) = \beta_1 + \beta_2 \times BI_t + \sum_{j=3}^{n} \beta_j \times CV_j + \vartheta_t$$

$$\vartheta_t = \rho \times \vartheta_{t-1}$$

Where,

μ_t: $average\ number\ of\ Aedes\ aegypti\ adult\ female\ mosquitoes\ in\ t$

t: $1\ to\ 39\ months$

β_1: $intercept$

$\beta_{2\ to\ n}$: $regression\ coefficients$

BI_t: $Breteau\ index\ in\ t$

CV_{jt}: $j\ (3\ to\ n) climate\ variable\ in\ t$

ϑ_t: $AR1\ temporal\ random\ effect$

ρ: $autoregressive\ parameter$

2 – Spatiotemporal model

2.1 Intercept model with random effects

$$Adult\ mosquitoes_{ti} = Poisson(\mu_{ti})$$

$$log(\mu_{ti}) = \beta_1 + v_{ti} + \omega_i$$

$$v_{ti} = \rho \times v_{t-1,i} + u_{ti}$$

Where,

μ_{ti}: $average\ number\ of\ Aedes\ aegypti\ adult\ female\ mosquitoes\ in\ t\ and\ i$

t: $1\ to\ 13\ quater - years\ representing\ the\ season\ of\ each\ year\ of\ the\ study\ period$

i: $1\ to\ 59\ adult\ traps\ in\ the\ households$

β_1: $intercept$

v_{ti}: $spatial\ random\ effect\ correlated\ temporally$

u_{ti}: $spatially\ correlated\ random\ effect$

ρ: $autoregressive\ parameter$

ω_i: $independent\ and\ identically\ distributed\ random\ effect$

2.2 Model with covariates

$$Adult\ mosquitoes_{ti} = Poisson(\mu_{ti})$$

$$log(\mu_{ti}) = \beta_1 + \beta_2 \times BI_{ti} + \sum_{j=3}^{n} \beta_j \times CV_{jt} + v_{ti} + \omega_i$$

$$v_{ti} = \rho \times v_{t-1,i} + u_{ti}$$

Where,

μ_{ti}: $average\ number\ of\ Aedes\ aegypti\ adult\ female\ mosquitoes\ in\ t\ and\ i$

t: $1\ to\ 13\ quater - years\ representing\ the\ season\ of\ each\ year\ of\ the\ study\ period$

i: $1\ to\ 59\ adult\ traps\ in\ the\ households$

β_1: $intercept$

$\beta_{2\ to\ n}$: $regression\ coefficients$

BI_{ti}: $Breteau\ index\ of\ the\ 150m\ buffer\ in\ quater - year\ t\ and\ trap\ i$

CV_{jt}: $j\ (3\ to\ n) climate\ variable\ in\ t$

v_{ti}: $spatial\ random\ effect\ correlated\ temporally$

u_{ti}: $spatially\ correlated\ random\ effect$

ρ: $autoregressive\ parameter$

ω_i: $independent\ and\ identically\ distributed\ random\ effect$
