# Supplementary material for: Association between densities of adult and immature stages of Aedes aegypti mosquitoes in space and time: implications for vector surveillance
Source: Parasit Vectors. 2022 Apr 19;15:133. doi: 10.1186/s13071-022-05244-4 (PMC9020056; doi:10.1186/s13071-022-05244-4)
Supplement: Supplementary file 5 — Additional file 5. Script for temporal model in R software. [file 13071_2022_5244_MOESM5_ESM.pdf]

Supplementary material 5 - Script for temporal model in R software

```
#### Temporal models

## packages

library(INLA)
library(INLAOutputs)
library(ggplot2)
library(lattice)

## database

bd <- read.csv("SupplementaryMaterial_1_Temporal_model_database.csv",sep=";")

## standardization of the covariates

bd.pdr <- bd
bd.pdr[,c(5:12)] <- scale(bd[,c(5:12)],scale =T)

########

## Intercept model

mod.I <- inla(mosq_ad~1,
        control.compute=list(dic=T),
        family = "poisson",
        data = bd)

mod.I$dic$dic
FixedEffects(mod.I)

# residuals

Pearson.residuals.Intercept.model <- (bd$mosq_ad - mod.I$summary.fitted.values$mean) /
sqrt(mod.I$summary.fitted.values$mean)
acf(Pearson.residuals.Intercept.model)

## Intercept + temporal random effect

mod.0.ar1 <- inla(mosq_ad~1 + f(ID, model = 'ar1'),
        control.compute=list(dic=T),
        family = "poisson",
        data = bd)

mod.0.ar1$dic$dic
FixedEffects(mod.0.ar1)

#residuals
```

```
Erro.0.ar1 <- (bd$mosq_ad - mod.0.ar1$summary.fitted.values$mean) /
sqrt(mod.0.ar1$summary.fitted.values$mean)
acf(Erro.0.ar1)

### temporal random effect

temp.random.effects_expon.AR1 <- lapply(mod.0.ar1$marginals.random$ID,
                        function(x) inla.emarginal(exp,x))

temp.random.effects_expon.unlist.AR1 <- unlist(temp.random.effects_expon.AR1)

##################################

# Models wiht covariates

## BI

mod.ib.ar1 <- inla(mosq_ad~1 + ib + f(ID, model = 'ar1'),
          control.compute=list(dic=T),
          family = "poisson",
          data = bd.pdr)

mod.ib.ar1$dic$dic
FixedEffects(mod.ib.ar1)

## residiuals

Pearson.residuals.Intercept.AR1.BI.model <- (bd.pdr$mosq_ad -
mod.ib.ar1$summary.fitted.values$mean) / sqrt(mod.ib.ar1$summary.fitted.values$mean)
acf(Pearson.residuals.Intercept.AR1.BI.model)

## Md_mn

mod.md.mn.ar1 <- inla(mosq_ad~1 + Md_mn + f(ID, model = 'ar1'),
            control.compute=list(dic=T),
            family = "poisson",
            data = bd.pdr)

mod.md.mn.ar1$dic$dic
FixedEffects(mod.md.mn.ar1)

## residiuals

Pearson.residuals.Intercept.AR1.MinTemp.model <- (bd.pdr$mosq_ad -
mod.md.mn.ar1$summary.fitted.values$mean) /
sqrt(mod.md.mn.ar1$summary.fitted.values$mean)
acf(Pearson.residuals.Intercept.AR1.MinTemp.model)
```

```
## precip

mod.prcp.ar1 <- inla(mosq_ad~1 + Prc_md_dia + f(ID, model = 'ar1'),
            control.compute=list(dic=T),
            family = "poisson",
            data = bd.pdr)

mod.prcp.ar1$dic$dic
FixedEffects(mod.prcp.ar1)

## residiuals

Pearson.residuals.Intercept.AR1.Precip.model <- (bd.pdr$mosq_ad -
mod.prcp.ar1$summary.fitted.values$mean) / sqrt(mod.prcp.ar1$summary.fitted.values$mean)
acf(Pearson.residuals.Intercept.AR1.Precip.model)

# IB and Md_mn

mod.ib.md.mn.ar1 <- inla(mosq_ad~1 + ib + Md_mn + f(ID, model = 'ar1'),
               control.compute=list(dic=T),
               family = "poisson",
               data = bd.pdr)

mod.ib.md.mn.ar1$dic$dic # 254.9
FixedEffects(mod.ib.md.mn.ar1)

## residiuals

Pearson.residuals.Intercept.AR1.BI.MinTemp.model <- (bd.pdr$mosq_ad -
mod.ib.md.mn.ar1$summary.fitted.values$mean) /
sqrt(mod.ib.md.mn.ar1$summary.fitted.values$mean)
acf(Pearson.residuals.Intercept.AR1.BI.MinTemp.model)

names(mod.ib.md.mn.ar1)

### temporal random effects

temp.random.effects_expon.mod.ib.md.mn.ar1 <- lapply(mod.ib.md.mn.ar1$marginals.random$ID,
                 function(x) inla.emarginal(exp,x))

temp.random.effects_expon.mod.ib.md.mn.ar1.unlist <-
unlist(temp.random.effects_expon.mod.ib.md.mn.ar1)

## IB and Precip

mod.ib.prc.ar1 <- inla(mosq_ad~1 + ib + Prc_md_dia + f(ID, model = 'ar1'),
               control.compute=list(dic=T),
               family = "poisson",
               data = bd.pdr)
```

```
mod.ib.prc.ar1$dic$dic
FixedEffects(mod.ib.prc.ar1)

## residiuals

Pearson.residuals.Intercept.AR1.BI.Precip.model <- (bd.pdr$mosq_ad -
mod.ib.prc.ar1$summary.fitted.values$mean) / sqrt(mod.ib.prc.ar1$summary.fitted.values$mean)
acf(Pearson.residuals.Intercept.AR1.BI.Precip.model)

# IB, Md_mn and prec

mod.ib.md.mn.prc.ar1 <- inla(mosq_ad~1 + ib + Md_mn + Prc_md_dia + f(ID, model = 'ar1'),
                    control.compute=list(dic=T),
                    family = "poisson",
                    data = bd.pdr)

mod.ib.md.mn.prc.ar1$dic$dic
FixedEffects(mod.ib.md.mn.prc.ar1)

## residiuals

Pearson.residuals.Intercept.AR1.BI.MinTemp.Precip.model <- (bd.pdr$mosq_ad -
mod.ib.md.mn.prc.ar1$summary.fitted.values$mean) /
sqrt(mod.ib.md.mn.prc.ar1$summary.fitted.values$mean)
acf(Pearson.residuals.Intercept.AR1.BI.MinTemp.Precip.model)
```
