# Supplementary material for: Association between densities of adult and immature stages of Aedes aegypti mosquitoes in space and time: implications for vector surveillance
Source: Parasit Vectors. 2022 Apr 19;15:133. doi: 10.1186/s13071-022-05244-4 (PMC9020056; doi:10.1186/s13071-022-05244-4)
Supplement: Supplementary file 6 — Additional file 6. Script for spatiotemporal model in R software. [file 13071_2022_5244_MOESM6_ESM.pdf]

Supplementary material 6 - Script for spatiotemporal model in R software

```
#### Spatiotemporal models

## packages

library(INLA)
library(tidyverse)
library(INLAOutputs)

## database

banco <- read.csv("SupplementaryMaterial_3_Spatiotemporal_model_database.csv",sep=";")

# standardizing the covariates

banco$BI.pdr <- scale(banco$BI,scale =T)
banco$Temp_Max.pdr <- scale(banco$Temp_Max,scale =T)
banco$Temp_Min.pdr <- scale(banco$Temp_Min,scale =T)
banco$Media_Max.pdr <- scale(banco$Media_Max,scale =T)
banco$Media_Min.pdr <- scale(banco$Media_Min,scale =T)
banco$Temp_Media.pdr <- scale(banco$Temp_Media,scale =T)
banco$Evapo_Media.pdr <- scale(banco$Evapo_Media,scale =T)
banco$Precip_Media_Dia.pdr <- scale(banco$Precip_Media_Dia,scale =T)

### Intercept model

I0 <- inla(adults~1,
        control.compute=list(dic=T),
        family = "poisson",
        data = banco)

FixedEffects(I0)

I0$dic$dic

## Models with spatial and iid random effects

Loc <- cbind(banco$LONG_UTM, banco$LAT_UTM)

# mesh

mesh <- inla.mesh.2d(Loc, max.edge=c(100,200),cutoff=1)

Ntrim <- length(unique(banco$ID.season))

####### PC priors

spde.pc.priors   <- inla.spde2.pcmatern(mesh, alpha = 2,
```

```
                                        prior.range=c(100, 0.5),
                                        prior.sigma=c(1, 0.5),
                                        constr=TRUE)

A1 <- inla.spde.make.A(mesh,
                       group = banco$ID.season,
                       loc = Loc)

w.index <- inla.spde.make.index(
  name    = 'w',
  n.spde  = spde.pc.priors$n.spde,
  n.group = Ntrim)

N <- nrow(banco)

###### Intercept and random effect model

X <- data.frame(Intercept = rep(1, N),
                ID = banco$ID)

Stk1 <- inla.stack(
  tag  = "Fit",
  data = list(y = banco$adults),
  A    = list(A1, 1),
  effects = list(
    w = w.index,
    X = as.data.frame(X)))

f1 <- y ~ -1 + Intercept +
  f(w,
    model = spde.pc.priors,
    group = w.group,
    control.group = list(model='ar1')) +
  f(ID,
    model = "iid",
    constr = TRUE)



I1 <- inla(f1,
        family = "poisson",
        data = inla.stack.data(Stk1),
        control.compute = list(dic = TRUE),
        control.predictor = list(A = inla.stack.A(Stk1)))


FixedEffects(I1)
I1$dic$dic
```

```
names(I1)

I1$marginals.random

###############################

#### Obtaining the spatial random effects for the mesh points and seasons (wis)

### wis - 13 seasons x 581 mesh vertices = 7553

w.pm  <- I1$summary.random$w$mean # wis non exponentiated

### wis exponentiated

w.pm.expon <- lapply(I1$marginals.random$w,
                 function(x) inla.emarginal(exp,x))

w.p.expon.unlist <- unlist(w.pm.expon)

## joining the wis with the mesh coordenates and seasons

long <- mesh$loc[,1]
lat <- mesh$loc[,2]

long.rep <- rep(long,13)
lat.rep <- rep(lat,13)

w.pm.exp.coord <- as.data.frame(cbind(long,lat,w.p.expon.unlist))

# creating a vector with the 13 seasons

trim <-
rep(c('a.su16','b.au16','c.wi16','d.sp16','e.su17','f.au17','g.wi17','h.sp17','i.su18','j.au18','k.wi18','l.sp18'
,'m.su19'),each=581)

w.pm.exp.coord$trim <- trim

ID <- rep(c(1:581),13)

w.pm.exp.coord$ID <- ID

wis.coord.trim <- w.pm.exp.coord %>%
  spread (key = trim ,  value = w.p.expon.unlist)

#######################################

#### using IDW to expand the wis for a grid of 100 x 100 points

# packages
```

```
require(gstat)
require(maptools)
require(sf)
require(spdep)

# identifying bounding

range(wis.coord.trim$long) # 671549.5 673515.3
range(wis.coord.trim$lat) # 7692703 7695088

gr=expand.grid(seq(671549.5,673515.3,len=100),seq(7692703,7695088,len=100))

head(gr)
grnovo=list()
grnovo=data.frame(long=gr$Var1,lat=gr$Var2)
head(grnovo)
class(grnovo)

### Prediction of the wis values for the grid points

# summer 2016
IDWch.a.su16 <- idw(a.su16~1,locations=~long+lat,data=wis.coord.trim, newdata=grnovo)
head(IDWch.a.su16)

# autumn 2016'
IDWch.b.au16 <- idw(b.au16~1,locations=~long+lat,data=wis.coord.trim, newdata=grnovo)
head(IDWch.b.au16)

# creating a database with all predicted wis

wis.pred.grid.trim <- IDWch.a.su16[,c(1:2)]
wis.pred.grid.trim$a.su16 <- IDWch.a.su16[,3]
wis.pred.grid.trim$b.au16 <- IDWch.b.au16[,3]

# winter 2016
IDWch.c.wi16 <- idw(c.wi16~1,locations=~long+lat,data=wis.coord.trim, newdata=grnovo)
head(IDWch.c.wi16)

wis.pred.grid.trim$c.wi16 <- IDWch.c.wi16[,3]

# spring 2016
IDWch.d.sp16 <- idw(d.sp16~1,locations=~long+lat,data=wis.coord.trim, newdata=grnovo)
head(IDWch.d.sp16)

wis.pred.grid.trim$d.sp16 <- IDWch.d.sp16[,3]

# summer 2017
IDWch.e.su17 <- idw(e.su17~1,locations=~long+lat,data=wis.coord.trim, newdata=grnovo)
```

```
head(IDWch.e.su17)

wis.pred.grid.trim$e.su17 <- IDWch.e.su17[,3]

# autumn 2017
IDWch.f.au17 <- idw(f.au17~1,locations=~long+lat,data=wis.coord.trim, newdata=grnovo)
head(IDWch.f.au17)

wis.pred.grid.trim$f.au17 <- IDWch.f.au17[,3]

# winter 2017
IDWch.g.wi17 <- idw(g.wi17~1,locations=~long+lat,data=wis.coord.trim, newdata=grnovo)
head(IDWch.g.wi17)

wis.pred.grid.trim$g.wi17 <- IDWch.g.wi17[,3]

# spring 2017
IDWch.h.sp17 <- idw(h.sp17~1,locations=~long+lat,data=wis.coord.trim, newdata=grnovo)
head(IDWch.h.sp17)

wis.pred.grid.trim$h.sp17 <- IDWch.h.sp17[,3]

# summer 2018
IDWch.i.su18 <- idw(i.su18~1,locations=~long+lat,data=wis.coord.trim, newdata=grnovo)
head(IDWch.i.su18)

wis.pred.grid.trim$i.su18 <- IDWch.i.su18[,3]

# autumn 2018
IDWch.j.au18 <- idw(j.au18~1,locations=~long+lat,data=wis.coord.trim, newdata=grnovo)
head(IDWch.j.au18)

wis.pred.grid.trim$j.au18 <- IDWch.j.au18[,3]

# winter 2018
IDWch.k.wi18 <- idw(k.wi18~1,locations=~long+lat,data=wis.coord.trim, newdata=grnovo)
head(IDWch.k.wi18)

wis.pred.grid.trim$k.wi18 <- IDWch.k.wi18[,3]

# spring 2018
IDWch.l.sp18 <- idw(l.sp18~1,locations=~long+lat,data=wis.coord.trim, newdata=grnovo)
head(IDWch.l.sp18)

wis.pred.grid.trim$l.sp18 <- IDWch.l.sp18[,3]

# summer 2019
IDWch.m.su19 <- idw(m.su19~1,locations=~long+lat,data=wis.coord.trim, newdata=grnovo)
head(IDWch.m.su19)
```

```
wis.pred.grid.trim$m.su19 <- IDWch.m.su19[,3]

ID <- seq(c(1:10000))

wis.pred.grid.trim$ID <- ID

## We exported this database and used the QGIS software to obtain the maps of the exponentiated spatial random effects for each season

##############################################

### Obtainning the models with the covariates

##### Breteau index

X2 <- data.frame(Intercept = rep(1, N),
                 ID = banco$ID,
                BI.pdr = banco$BI.pdr)



#Stack
Stk2 <- inla.stack(
  tag  = "Fit",
  data = list(y = banco$adults),
  A    = list(A1, 1),
  effects = list(
    w = w.index,
    X = as.data.frame(X2)))

f2 <- y ~ -1 + Intercept + BI.pdr +
  f(w,
    model = spde.pc.priors,
    group = w.group,
    control.group = list(model='ar1')) +
  f(ID,
    model = "iid",
    constr = TRUE)

I2 <- inla(f2,
        family = "poisson",
        data = inla.stack.data(Stk2),
        control.compute = list(dic = TRUE),
        control.predictor = list(A = inla.stack.A(Stk2)))

FixedEffects(I2)
I2$dic$dic
### valores novos
```

```
# mean qt0.025 qt0.975
# Intercept 0.870   0.701   1.061
# IB.pdr    1.117   0.996   1.244

 # 2266.732

### valores antigos

### rodando extamente igual ao livro

#           mean qt0.025 qt0.975
#Intercept 0.872   0.701   1.068
#IB.pdr    1.125   1.004   1.253

I2$dic$dic # 2270.1

########

#### including the climatic variable in the models

## BI and average minimum temperature

X2.ib.med.mn <- data.frame(Intercept = rep(1, N),
                          ID=banco$ID,
                 BI.pdr = banco$BI.pdr,
                 Media_Min.pdr=banco$Media_Min.pdr)



#Stack
Stk2.ib.med.mn <- inla.stack(
  tag  = "Fit",
  data = list(y = banco$adults),
  A    = list(A1, 1),
  effects = list(
    w = w.index,            #Spatial field
    X = as.data.frame(X2.ib.med.mn)))  #Covariates

f2.ib.med.mn <- y ~ -1 + Intercept + BI.pdr + Media_Min.pdr +
  f(w,
    model = spde.pc.priors,
    group = w.group,
    control.group = list(model='ar1')) +
  f(ID,
    model = "iid",
    constr = TRUE)

I2.ib.med.mn <- inla(f2.ib.med.mn,
          family = "poisson",
          data = inla.stack.data(Stk2.ib.med.mn),
```

```
          control.compute = list(dic = TRUE),
          control.predictor = list(A = inla.stack.A(Stk2.ib.med.mn)))

FixedEffects(I2.ib.med.mn)

I2.ib.med.mn$dic$dic

## average minimum temperature

X2.med.mn <- data.frame(Intercept = rep(1, N),
                    ID=banco$ID,
                      Media_Min.pdr=banco$Media_Min.pdr)



#Stack
Stk2.med.mn <- inla.stack(
  tag  = "Fit",
  data = list(y = banco$adults),
  A    = list(A1, 1),
  effects = list(
    w = w.index,           #Spatial field
    X = as.data.frame(X2.med.mn)))  #Covariates

# modelo com autocorr espacial e autorregressiso (no livro chamou apenas de autorreg)

f2.med.mn <- y ~ -1 + Intercept + Media_Min.pdr +
  f(w,
    model = spde.pc.priors,
    group = w.group,
    control.group = list(model='ar1')) +
  f(ID,
    model = "iid",
    constr = TRUE)

I2.med.mn <- inla(f2.med.mn,
              family = "poisson",
              data = inla.stack.data(Stk2.med.mn),
              control.compute = list(dic = TRUE),
              control.predictor = list(A = inla.stack.A(Stk2.med.mn)))

FixedEffects(I2.med.mn)

I2.med.mn$dic$dic

### BI and precipitation

X2.ib.prec <- data.frame(Intercept = rep(1, N),
                      BI.pdr = banco$BI.pdr,
                     ID=banco$ID,
```

```
                    Precip_Media_Dia.pdr=banco$Precip_Media_Dia.pdr)


#Stack
Stk2.ib.prec <- inla.stack(
  tag  = "Fit",
  data = list(y = banco$adults),
  A    = list(A1, 1),
  effects = list(
    w = w.index,
    X = as.data.frame(X2.ib.prec)))

f2.ib.prec <- y ~ -1 + Intercept + BI.pdr + Precip_Media_Dia.pdr +
  f(w,
    model = spde.pc.priors,
    group = w.group,
    control.group = list(model='ar1')) +
  f(ID,
    model = "iid",
    constr = TRUE)

I2.ib.prec <- inla(f2.ib.prec,
                   family = "poisson",
                   data = inla.stack.data(Stk2.ib.prec),
                   control.compute = list(dic = TRUE),
                   control.predictor = list(A = inla.stack.A(Stk2.ib.prec)))

FixedEffects(I2.ib.prec)
I2.ib.prec$dic$dic

#### precipitation

X2.prec <- data.frame(Intercept = rep(1, N),
                    ID=banco$ID,
                      Precip_Media_Dia.pdr=banco$Precip_Media_Dia.pdr)

#Stack
Stk2.prec <- inla.stack(
  tag  = "Fit",
  data = list(y = banco$adults),
  A    = list(A1, 1),
  effects = list(
    w = w.index,
    X = as.data.frame(X2.prec)))

f2.prec <- y ~ -1 + Intercept + Precip_Media_Dia.pdr +
  f(w,
    model = spde.pc.priors,
    group = w.group,
```

```
    control.group = list(model='ar1')) +
  f(ID,
    model = "iid",
    constr = TRUE)

I2.prec <- inla(f2.prec,
               family = "poisson",
               data = inla.stack.data(Stk2.prec),
               control.compute = list(dic = TRUE),
               control.predictor = list(A = inla.stack.A(Stk2.prec)))

FixedEffects(I2.prec)
I2.prec$dic$dic

# BI, average minimum temperature and precipitation

X2.ib.med.mn.prec <- data.frame(Intercept = rep(1, N),
                         ID=banco$ID,
                      BI.pdr = banco$BI.pdr,
                      Media_Min.pdr=banco$Media_Min.pdr,
                      Precip_Media_Dia.pdr=banco$Precip_Media_Dia.pdr)



#Stack
Stk2.ib.med.mn.prec <- inla.stack(
  tag  = "Fit",
  data = list(y = banco$adults),
  A    = list(A1, 1),
  effects = list(
    w = w.index,
    X = as.data.frame(X2.ib.med.mn.prec)))

f2.ib.med.mn.prec <- y ~ -1 + Intercept + BI.pdr + Media_Min.pdr + Precip_Media_Dia.pdr +
  f(w,
    model = spde.pc.priors,
    group = w.group,
    control.group = list(model='ar1')) +
  f(ID,
    model = "iid",
    constr = TRUE)

I2.ib.med.mn.prec <- inla(f2.ib.med.mn.prec,
                family = "poisson",
                data = inla.stack.data(Stk2.ib.med.mn.prec),
                control.compute = list(dic = TRUE),
                control.predictor = list(A = inla.stack.A(Stk2.ib.med.mn.prec)))

FixedEffects(I2.ib.med.mn.prec)
I2.ib.med.mn.prec$dic$dic
```
